# Improving the Hydraulic Effects Resulting from the Use of a Submerged Biofiter to Enhance Water Quality in Polluted Streams

**DOI:** 10.3390/ijerph182312351

**Published:** 2021-11-24

**Authors:** Atef A. El-Saiad, Hany F. Abd-Elhamid, Zeinab I. Salama, Martina Zeleňáková, Erik Weiss, Emad H. El-Gohary

**Affiliations:** 1 Department of Water and Water Structures Engineering, Faculty of Engineering, Zagazig University, Zagazig 44519, Egypt; aelsaiad123@yahoo.com; 2 Center for Research and Innovation in Construction, Faculty of Civil Engineering, Technical University of Košice, 04200 Košice, Slovakia; 3 Higher Institute of Engineering and Technology, Zagazig 44519, Egypt; zeinab.salama92@yahoo.com; 4 Department of Environmental Engineering, Faculty of Civil Engineering, Technical University of Košice, 04200 Košice, Slovakia; 5 Department of Commercial Entrepreneurship, Faculty of Business Economy with Seat in Kosice, University of Economics in Bratislava, 04001 Košice, Slovakia; erik.weiss@euba.sk; 6 Environmental Engineering Department, Faculty of Engineering, Zagazig University, Zagazig 44519, Egypt; dr_abohamdy@yahoo.com

**Keywords:** water quality, polluted streams, submerged biofilter (SB), relative heading up, hydraulic properties

## Abstract

Water scarcity is one of the most serious problems facing many countries. In addition, water pollution could lose more water. A submerged biofilter (SB) is used to enhance the self-purification process in polluted streams. However, most previous studies have focused on the efficiency of SB to remove pollutants and there is a lack of studies investigating the hydraulic changes in streams. The current paper aimed to study the hydraulic effects of SB on the flow behavior in streams and how to improve it. An empirical equation for determining the flow rate through SB was developed. Different cases were studied to improve the hydraulic effects resulting from the use of SB. The effect of increasing SB length was tested using different SB lengths. The results showed that increasing the length increased the upstream water depth (h_1_) and relative heading up (h_1_/h_2_). In the second case, comparison between continuous and fragmented SB was tested. The results showed that a fragmented biofilter increased the upstream water depth and the relative heading up. Case three tested the effect of SB height. Different SB heights were tested with a fixed length and constant flow rate. The results revealed that the upstream water depth and relative heading up decreased when the biofilter height decreased. Case four tested the effect of SB with a fixed volume and constant flow rate. In this case, the length and height of SB were changed where the volume was fixed. The results showed that the relative heading up decreased when the SB height decreased and the length increased, which revealed that the SB height can improve the hydraulic impacts. Finally, the use of SB to improve the water quality in polluted streams led to an increase of the relative heading up, which can be reduced by decreasing the height of SB.

## 1. Introduction

Water is a vital natural resource that is essential for a multiplicity of purposes. Egypt depends on the Nile River to secure nearly 95% of the water needed for different purposes, such as drinking, household uses, agriculture, fishing, transportation, tourism, and electricity generation [1]. Rainfall is very rare, except for a very small strip along the coast of the Mediterranean. Groundwater is available in parts of the Western and Eastern Deserts and Sinai. Egypt’s municipal, agricultural, and industrial water requirements have increased with time due to the increase in population and the improvement of living standards [2]. By 2025, the demand of water for agricultural purposes will increase by 60% and the percentage of water used for industrial purposes will be about 20% and 10% of the total for domestic purposes [3]. Egypt has limited water resources due to its fixed share of the Nile water. Lake Nasser represents the national freshwater bank of Egypt, depending on the water inflow through the Nile [4]. In April 2011, Ethiopia launched the construction of the Grand Ethiopian Renaissance Dam (GERD), Bameza, Ethiopia, with a water storage capacity of 74 billion m^3^ that may cut into Egypt’s share of the Nile. Egyptian experts predicted a water reduction of about 20–34% owing to the construction of the Ethiopian Dam. This was estimated to be 11–19 billion m^3^ on average over the dam’s filling period [5]. Consequently, about one-third of the total agricultural lands may be subjected to drought [6]. This creates a major concern for Egypt, with concerns that this dam will decrease it’s share from the Nile River (55.5 billion m^3^/year). Egypt is concerned that during dry months, not enough water will be released from the GERD, thus decreasing the water received downstream [7].

There is a tendency to use unconventional water resources, such as desalinated water, which is high cost compared to other water resources. The cost of desalting 1 m^3^ of sea water ranged between 5.40 and 18.88 Egyptian pound (LE) depending on the capacity of the desalination plant, which makes use of this water for agricultural purposes, which is economically inefficient at present [6,7,8]. So, the Egyptian government is going to treat wastewater to protect environmental resources, such as soil, groundwater, and water reservoirs. Treated wastewater (TWW) is a reliable water source that can fulfill the gap between water demand and supply, but the use of untreated wastewater without control in agriculture will affect the groundwater quality [3]. In Egypt, TWW can contribute up to 5 × 10^9^ m^3^/year to water resources. Current TWW use is about 1.3 × 10^9^ m^3^/year, which can be expanded to around 3 × 10^9^ m^3^/year, of secondary treated wastewater. Addressing the social and management challenges as well as the environmental and heath aspects of recycling TWW is essential before implementing large-scale TWW projects [9].

The reuse of agricultural drainage water is a common practice in Egypt due to water scarcity, although some of these agricultural drains turn into major carriers of untreated wastewater, which is subsequently utilized for irrigation [10]. Moreover, the absence of sanitation systems in the rural Nile delta drives farmers to discharge wastewater into agricultural drains [11]. In order to use agricultural drainage water in agriculture, the water quality should be enhanced to meet the national standards. One of the proposed methods to enhance the drain water quality is the use of SB. A number of researchers proposed different approaches to assess the use of SB to enhance the water quality in polluted drains. Abd El-Rahman [12] studied the use of SB for reducing the organic content from rivers polluted with domestic wastes. He used three different plastic media (plate settler, tube settler, and plastic balls) as biofilters. Ramírez et al. [13] stated that SB is a low-cost system that will enhance the water quality in small polluted rivers and can be constructed in situ. During his study, two stream models were constructed, and both streams were filled with crushed river stone. A mixture of primary and secondary effluents from a wastewater treatment plant (WWTP) was used to test the models. The results showed that the chemical oxygen demand (COD) removal efficiency increased in streams packed with the media.

Moussa et al. [14] studied the self-purification capacity of Muheit and Rahawy drains in Egypt. They used anaerobic biofilm reactors by hanging submerged plastic packing media in the drain to increase the waste assimilative capacity of these drains. El Monayeri et al. [15,16] studied the effect of using submerged media (pall rings—star shape—gravel) in four pilot streams on the performance of the biological degradation process and the hydraulic scheme of streams in Egypt. The results showed that the media (pall rings and star shape) are more effective in the removable of the biochemical oxygen demand (BOD) and COD than gravel, while the pall rings media had the lowest effect on the hydraulic scheme of the canal’s water flow. El Monayeri et al. [17] studied the use of submerged biofilters in polluted streams for increasing the self-purification capacity at the discharging point. The results showed that the performance of SB is affected by the total hydraulic loading (THL). As the THL increases, the COD removal ratio decreases and the pall rings media biofilter achieved the highest COD removal ratio compared to the star shape and gravel while providing the same total surface area for biofilm growth.

He et al. [18] studied the performance and nitrobacteria population dynamics in a pilot-scale SB for landscape river water purification during a startup period of over 45 days. The general nitrobacteria population coupled with the performance of SB indicated that the dynamics of nitrobacteria was an important indicator to evaluate the effect of the biofilter system. Abdel Daiem et al. [19] studied the radial basis function neural network (one conventional and one based on particle swarm optimization (PSO) are employed) to accurately predict the removal of COD from polluted streams using SB media (plastic and gravel) under the influence of different variables, such as the temperature, flow rate, and influent COD. The results showed that the COD removal ratio had the highest value (65%) when two plastic biofilter media were used at the minimum flow rate. Salem [20] investigated the concentrations of heavy metals and pesticides in wastewater from three main drains in Egypt. The results showed that the concentrations of some heavy metals exceeded their respective permissible limits in water samples.

According to the previous literature, most previous studies have focused on the efficiency of the biofilter to remove pollutants from water streams and there is a lack of studies on the hydraulic changes in the streams, such as increasing the water level upstream and relative heading up. The relative heading up of the water surface in water streams may cause some effects on the groundwater table or embankments, or may deteriorate the agricultural drainage network. Fadhil et al. [21] conducted a study to investigate the flow through and over gravel gabion weirs (GGWs), which consist of the gabion length and the height of the gabion on its upstream water depth. The results showed that for a through flow regime for the same gravel size, the upstream water depth of the gabion increased by increasing the weir length [21,22]. The current study aimed to assess the hydraulic effects of the SB on water streams. These biofilters result in probable variations in flow behavior, such as the upstream heading up. During this study, the hydraulic conductivity of the used medium (Kc) was determined. It is possible to obtain a mathematical formula from which to predict the relative heading up of the water surface in streams by knowing the specifications of the biofilter used and the width (B), water depth (h), and the flow rate (Q) of the water body. Different scenarios to assess the impact of SB characteristics on the hydraulic properties of the stream were tested. The effects of the biofilter length (L) in the flow direction, and the flow rate (Q) on the relative heading up (h_1_/h_2_) were studied. The effect of using a fragmented biofilter in the flow direction on the relative heading up (h_1_/h_2_) was studied and compared with a continuous biofilter. The effect of the biofilter height on the relative heading up (h_1_/h_2_) at a fixed length and flow rate was studied. Additionally, the effect of the biofilter length and height with a fixed volume on the relative heading up (h_1_/h_2_) was tested.

## 2. Materials and Methods

### 2.1. Description of the Experimental Work

**Figure 1:**
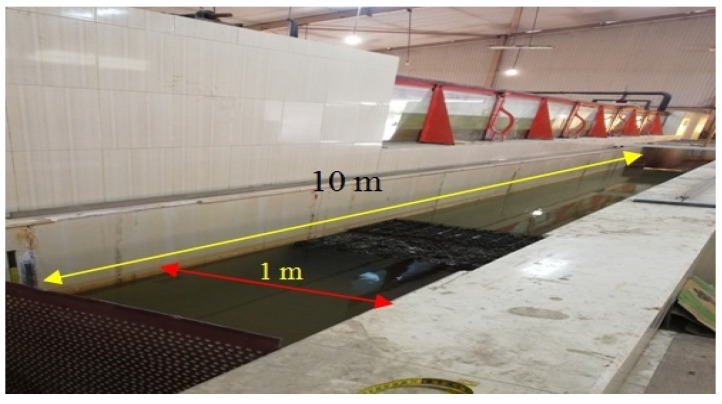
Canal used in the experimental study.

**Figure 2:**
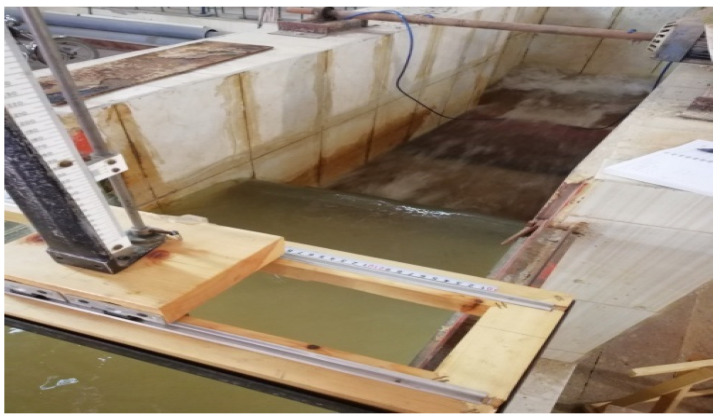
General view of the calibrated weir.

**Figure 3:**
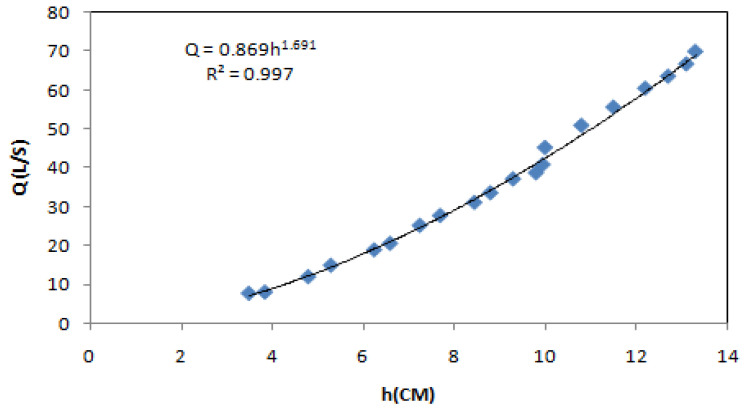
Calibration curve of the rectangular weir.

The experimental work was carried out using a canal of 1.0 m width, 10 m length, and 1.0 m depth as shown in Figure 1. This canal is provided with a pump with a varied flow rate (2 to 70 L·s^−1^) and rulers graded for measuring the water depth upstream and downstream of the horizontal biofilter, which was filled with plastic media (star shape). The inlet discharge to the canal was controlled using a rectangular weir (see Figure 2), and the discharge passing to the canal was determined by measuring the head of water (h) above the weir, which was calibrated in the lab as shown in Figure 3.

In order to investigate the hydraulic effects caused by the biofilter on the flow, the hydraulic conductivity (Kc) of the biofilter and the effect of using (SB) on the relative heading up were calculated at different discharges. The discharge equation obtained for the weir can be expressed as [15,16]:

Q = 0.869 ∗ h^1.69^ (1)

where Q is the discharge passing over the weir crest (L·s^−1^) and h is the head over the crest of the weir (cm).

### 2.2. Description of the Submerged Biofilter

**Figure 4:**
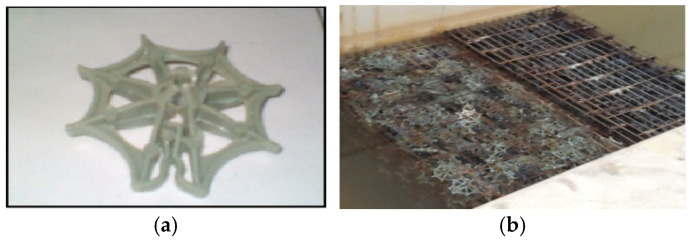
The plastic media used in this study (star shape). (**a**) Star shape media, (**b**) The biofilter.

The performance of the boifilter depends upon the type of media used and its characteristics, such as the specific surface area, void ratio, surface roughness, geometry, and configuration. During this work, the random packed media was selected and the used biofilter was star shaped. It has a high specific surface area of about 175.7 m^2^/m^3^, and a void ratio of about 87%. The media was placed in boxes (1.0 m length, 0.4 m width, 0.26 m height) as shown in Figure 4 and then installed in the stream according to the experimental cases [16,20].

### 2.3. Experimental Program

**Figure 5:**
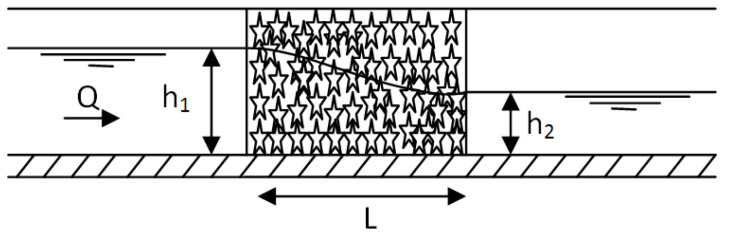
Sectional elevation in the canal where star media was installed.

During this study, the biofilter length (L) was changed from 0.4 to 2 m under varied flow rates for each length. At each flow rate, the water depths both upstream (h_1_) and downstream (h_2_) were measured. Figure 5 shows the section elevation in the canal where star media was installed, and Table 1 presents the operational conditions applied through the experimental study. For all the cases, the relative heading up (h_1_/h_2_) was calculated to show the effect of the submerged biofilter on the hydraulic properties of the stream.

## 3. Empirical Equation of the Flow Rate through the Submerged Biofilter

Different equations can be used to represent the flow rate (Q) through SB. In this part, the flow rate measured in the experimental study is compared with three empirical equations to select the most appropriate equation to be applied in calculating the flow rate through the submerged filter.

### 3.1. Dupuit Formula for Flow through Horizontal Filters

Khalifa et al. [23] studied the effect of the installation of porous media working as biofilters on the flow behavior in open canals. They derived an equation that relates the amount of flow passing through the media and hydraulic conductivity of the media, which is known as Dupuit formula, as follows [23]:

(2) $$Q=\frac{K_{c}b(h_{1}^{2}-h_{2}^{2})}{2L}$$

where Q = volumetric flow rate (m^3^·s^−1^), K_c_ = hydraulic conductivity of the medium (m·s^−1^), b = width of the biofilter (m), h_2_ = depth of water upstream the biofilter (m), h_2_ = depth of water downstream the biofilter (m), and L = length of the biofilter in the flow direction (m).

Data fit software is used to obtain the empirical equation for the measured experimental data (Q, b, h_1_, h_2_, L) presented in Equation (2) as follows [22,23]:

(3) $$y=\frac{ax_{1}(x_{2}^{2}-x_{3}^{2})}{2x_{4}}$$

The value of coefficient (a) is calculated which refers to the hydraulic conductivity of the medium (K_c_) = 1.57 m·s^−1^. The correlation coefficient R^2^ is 0.81 [24]. Using the Excel program and the value of K_c_ obtained from the empirical solution, the flow rate is calculated from this empirical equation:

(4) $$Q=\frac{1.57b(h_{1}^{2}-h_{2}^{2})}{2L}$$

### 3.2. Flow through Gravel Gabion Dams

Fadhil et al. [21] developed an empirical equation for computing the upstream water depth for the case of flow through gabion dams (or weirs). The developed equation was found with the correlation coefficient (R^2^ = 0.94), which can be expressed as follows [21]:

(5) $$h_{1}=1.607\frac{q^{0.66}L^{0.349}}{d_{m}{}^{0.34}}$$

where h_1_ = upstream water depth (m), q = discharge per unit width of the flume (unit discharge Q/b) (m^3^·s^−1^·m^−1^), L = gabion length (m), and d_m_ = mean gravel size used in gabion construction (m).

The Fadhil equation relates the flow rate through the gabion dam (Q) to the dam length (L) and width (B), neglecting the effect of the water depth downstream of the dam (h_2_). According to this equation, the flow rate is expressed as follows [21,22]:

(6) $$Q=a\frac{h_{1}{}^{b}B^{}}{L^{c}}$$

Data fit software is used to obtain empirical equation for the measured experimental data (Q, h_1_, B, L) presented in Equation (6) as follows [21]:

(7) $$Y=aX_{1}^{b}{}^{}X_{2}X_{3}^{c}$$

The values of coefficients a, b, and c are determined to be 0.398, 4.494, and −0.193. The correlation coefficient R^2^ = 0.966. Using the Excel program and the values of coefficients a, b, and c obtained from the empirical model, the flow rate is calculated from this empirical equation [21]:

(8) $$Q=0.398\frac{h_{1}{}^{4.494}B^{}}{L^{0.193}}$$

### 3.3. Flow through Horizontal Biofilters (Modified Fadhil’s Equation)

In the modified Fadhil’s method, it is suggested to replace the value of h_1_ in the Fadhil equation by the relative heading up (h_1_/h_2_). This is to test the strength of the relationship between the flow rate passing through the gabion dam with the relative heading up (h_1_/h_2_) compared to the relationship of the flow rate with the height of the water in front of the dam only (h_1_). The modified Fadhil equation is expressed as follows [21]:

(9) $$Q=a\frac{{\left(h_{1}/h_{2}\right)}^{b}B^{}}{L^{c}}$$

Data fit software is used to obtain the empirical equation for the measured experimental data (Q, h_1_/h_2_, B, L) presented in Equation (9) as follows [21]:

(10) $$Y=aX_{1}^{b}{}^{}X_{2}X_{3}^{c}$$

The values of coefficients a, b, and c are calculated as 1.64, 12.14, and −0.675. The correlation coefficient is R^2^ = 0.89. Using the Excel program and the value coefficients a, b, and c of obtained from the empirical model, the flow rate is calculated from this empirical equation:

(11) $$Q=1.64*\frac{{\left(\frac{h_{1}}{h_{2}}\right)}^{12.14}B^{}}{L^{0.675}}$$

### 3.4. Selection of the Proper Equation to Calculate the Flow Rate through the Submerged Biofilter

During the present work, the stream was packed with media at a height of (0.26 × 3 = 0.78 m) and different lengths, which gave different flow rates through the media. The total number of runs were 30 runs and the flow experiments are presented in Table 2. The table shows the obtained results for water depths upstream (h_1_) and downstream (h_2_), the resulting relative heading up (h_1_/h_2_), and the flow rate obtained by the three empirical Equations (4), (8), and (11).

**Figure 6:**
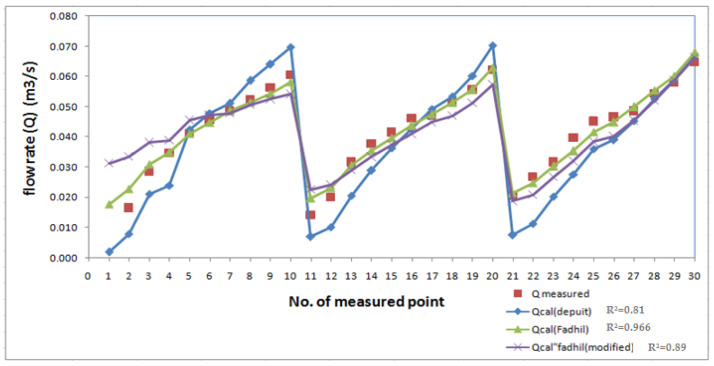
Comparison between the flow rate obtained by the empirical equations of Dupuit, Fadhil, and modified Fadhil and the experimental results.

The results of the experimental study for 30 runs were compared with the flow rate obtained from the three empirical Equations (4), (8), and (11) as shown in Table 2. Figure 6 shows the result of the calculated flow rates passing through the biofilter using the three equations compared to the experimental measured values. The equation of the flow through the gabion dam (Fadhil formula) is the closest representation to the submerged biological reactor used to improve polluted water streams compared to the other formulas as shown in Figure 6. It is clear that the flow rate passing through the biofilter depends on the height of the water in front of it (h_1_) more than on the relative heading up (h_1_/h_2_) (modified Fadhil’s equation). This study recommends using Equation (8) (Fadhil formula) to estimate the flow rate through the submerged filter (plastic star shape).

## 4. The Effect of the Biofilter Characteristics on the Hydraulic Properties of the Stream

Using SB in water streams (or drains) affected the hydraulic properties of the stream, such as the relative heading up. The installation of these biofilters in the stream should result in probable variations in the flow behavior. The most important variation is the relative heading up. The relative heading up of the water surface in water streams (or drains) may affect the groundwater table or embankments, or may deteriorate the agricultural drainage network. This part presents the effect of changing some characteristics of the biofilter and their impact on the hydraulic properties of the stream.

### 4.1. Effect of the Biofilter Length on the Relative Heading Up

**Figure 7:**
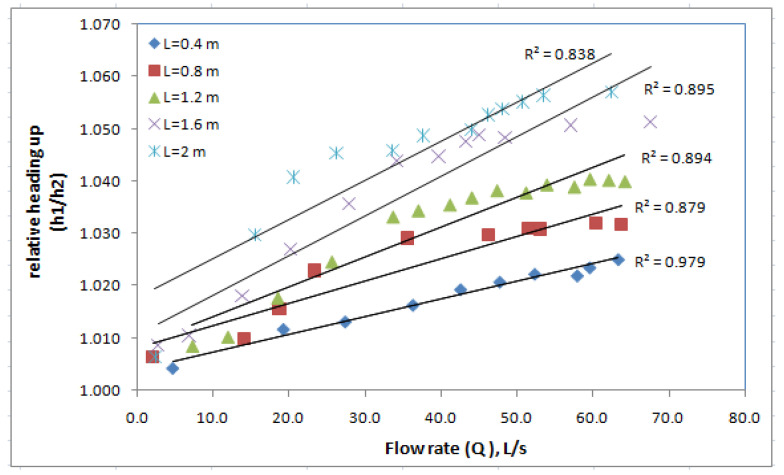
The relation between the relative heading up and flow rate passing through the biofilter at different lengths.

During this case, the stream was packed with media at a height (0.26 × 2 = 0.52 m) and the applied flow passed through and over the media. The measured water depths upstream (h_1_) and downstream (h_2_) and the resulting relative heading up (h_1_/h_2_) for different lengths of the biofilter are presented in Table 3. Additionally, the flow rate was measured for each case. Figure 7 shows the relation between the relative heading up and the flow rate passing through the biofilter at different lengths (0.4, 0.8, 1.2, 1.6, 2 m). The results show that the relation between the relative heading up (h_1_/h_2_) and flow rate (Q) is linear for all lengths. For the same discharge, the relative heading up (h_1_/h_2_) value increased with the increase of the length of the biofilter. As shown in Figure 7, L = 2 m gave the highest values of the relative heading up, which ranged from 1.006 to 1.057 when the flow rates increased from 2.3 to 62.5 L·s^−1^, followed by (L = 1.6 m), for which the relative heading up ranged from 1.009 to 1.051 when the flow rates increased from 2.8 to 67.6 L·s^−1^. For L = 1.2 m, the relative heading up ranged from 1.008 to 1.04 when the flow rates increased from 7.2 to 62.1 L·s^−1^, (L = 0.8 m) and the relative heading up ranged from 1.007 to 1.032 when the flow rates increased from 2 to 63.7 L·s^−1^. However, for L = 0.4 m, the relative heading up ranged from 1.004 to 1.025 when the flow rates increased from 4.7 to 63.3 L·s^−1^ [21,25].

### 4.2. Effect of Using a Fragmented Biofilter in the Flow Direction on the Relative Heading Up

The effect of dividing the biofilter in the flow direction is examined in this stage including two cases: the continuous biofilter and fragmented biofilter. Muslu (1986) proposed the following equation for the removal efficiency of SB [26]:

(12) $$L_{e}/L_{i}=e^{-\beta H}$$

where L_i_ = the influent concentration of COD at the biofilter (mg/L), L_e_ = the expected concentration of COD at the effluent from the biofilter (mg/L), β = factors depending on the used media proprieties and applied discharge (m^−1^), and H = length of the biofilter in the flow direction (m).

From Equation (12), it is clear that the relationship between the COD removal ratio and the length of the biofilter is nonlinear but rather exponential. This means that if the length of the reactor is 1 m and the β value is 0.2 (for example), then the removal ratio is 0.82, but when the length of the reactor is doubled to 2 m, the removal ratio does not double as well but becomes 0.67. This means that from the mathematical point of view, it is preferable to use successive small length reactors to obtain a higher percentage of removal than using one long continuous reactor. However, the effect of dividing the biofilter into several successive reactors on the flow behavior is not known.

**Figure 8:**
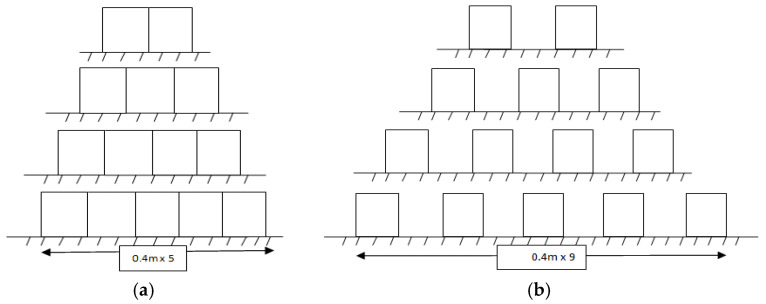
Sectional elevation in the canal where a biofilter was installed as continuous and fragmented in the flow direction. (**a**) Continuous biofilter, (**b**) Fragmented biofilter.

In this case, the experimental work was carried out in two subsequent cases. The first case was conducted on a continuous biofilter in the flow direction and the second case was carried out on a fragmented biofilter (the distance between the biofilter is equal to the length of the biofilter in the flow direction) as shown in Figure 8. In each case, the height of the SB is 0.26 × 2 = 0.52 m with a varied total length (0.8, 1.2, 1.6, and 2.0 m). The upstream water depth (h_1_) and downstream water depth (h_2_) were measured and the relative heading up (h_1_/h_2_) was calculated in the two cases for each length of the biofilter at a constant flow rate. Table 4 shows the obtained results by the continuous biofilter (case 1) and fragmented biofilter (case 2).

From the obtained results in the two cases, it was noticed that for a biofilter length L = 0.8 m, the relative heading up (h_1_/h_2_) in the fragmented biofilter was 1.031, which is higher than the continuous biofilter 1.029. At the length L = 1.2 m, the relative heading up (h_1_/h_2_) in the fragmented biofilter was 1.036 compared with the continuous biofilter, which was 1.033. At the length L = 1.6 m, the relative heading up (h_1_/h_2_) in the fragmented biofilter was 1.050 compared with 1.048 in the continuous biofilter and at the length L = 2.0 m, the relative heading up (h_1_/h_2_) in the fragmented biofilter was 1.057 compared with 1.052 in the continuous biofilter. The results indicated that the fragmented biofilter gave a higher relative heading up (h_1_/h_2_) compared to the results obtained from the continuous biofilter because the total length in the flow direction increased. The results of this case reveal that the continuous biofilter is more hydraulically efficient than the fragmented biofilter as it gave less relative heading up.

### 4.3. Effect of the Biofilter Height on the Relative Heading Up

**Figure 9:**
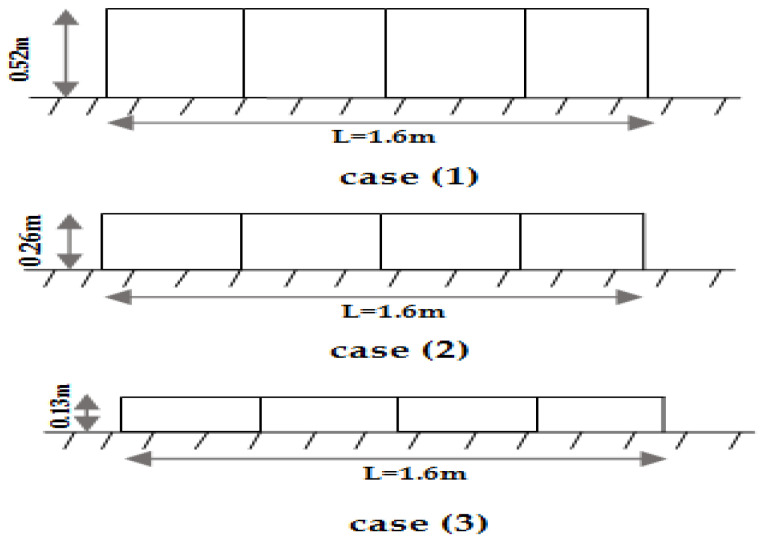
Sectional elevation in the canal where the biofilter was installed at different heights.

During this case, the biofilter height changed from 0.52 to 0.26 and 0.13 m and the length of the biofilter was constant (L = 1.6m) with a constant flow rate of 18.95 L·s^−1^. For each case, the water depth upstream of the biofilter was measured. Figure 9 shows the section elevation in the canal where the biofilter was installed and Table 5 presents the measured water depths upstream and downstream and the resulting relative heading up for each case.

The results obtained from operating the biofilters at different heights with a constant length and flow rate indicated that the relative heading up was 1.03 in case (1), followed by 1.009 in case (2), and it finally decreased to 1.0 in case (3). The results show that the relative heading up (h_1_/h_2_) decreased when the biofilter height decreased.

### 4.4. Effect of the Fixed Volume Biofilter with a Variable Length and Height on the Relative Heading Up

**Figure 10:**
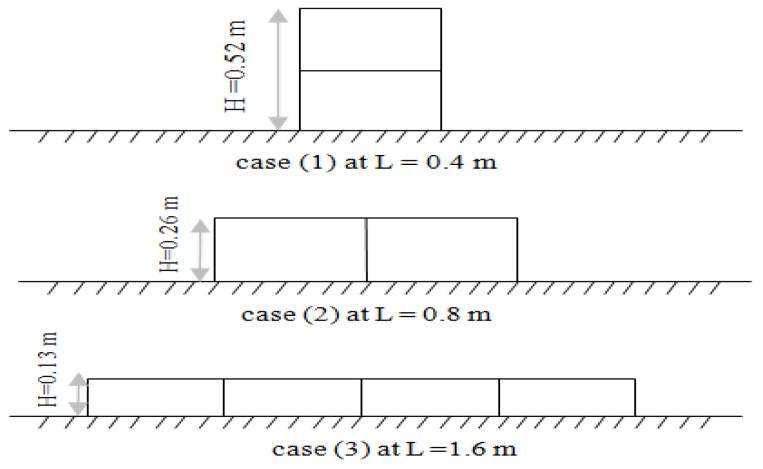
Sectional elevation in the canal where the biofilter was installed with a constant volume with variable length and height.

During this case, the biofilter length (L) changed to 0.4, 0.8, and 1.6 m and the height of the biofilter changed from 0.52 to 0.26 and 0.13 m to obtain a constant volume of media at a constant flow rate of 18.95 L·s^−1^. For each case, the water depths upstream and downstream were measured and the resulting relative heading up was determined. Figure 10 shows the section elevation in the canal where the biofilter was installed and Table 6 presents the measured water depths upstream and downstream of the biofilter for each case.

The results obtained from operating the biofilters with a fixed volume and constant flow rate with a variable length and height indicated that the relative heading up was 1.0096 in case (1), followed by 1.0019 in case (2), and finally the lowest value of 1 in case (3). Other studies considered that a fixed volume would perform better as the length in the flow direction is increased [27,28]. The most recent biological filter model predicts only a minor increase in performance with deeper towers of the same volume [29]. The current results showed that for a biofilter with a fixed volume and constant flow rate with variable length and height, the relative heading up decreased and improved the performance when the height of the biofilter decreased.

## 5. Discussion

The treatment and reuse of contaminated wastewater has become a crucial issue. In this study, SB was used to enhance the water quality in polluted streams. Using such filters enhanced the removal of pollutants from streams but affected the hydraulic characteristics of the waterway, such as increasing the water level upstream. This study focused on improving the hydraulic effects of the SB on flow in streams. An empirical equation was developed to determine the flow rate in the submerged biofilter. Three empirical equations (Depuit formula, Fadhil formula, and modified Fadhil formula) were used to predict the flow rate through the biofilter and compared with experimental results. The results showed that the equation of the flow through the gabion dam (Fadhil formula) is the closest representation of the SB with (R^2^ = 0.966) followed by the modified Fadhil formula (R^2^ = 0.89) and Depuit formula (R^2^ = 0.81). The results indicate that the flow rate through the biofilter depends on the height of the water in front of it (h_1_) more than on the relative heading up (h_1_/h_2_).

Based on the experimental program executed in this study, using the SB increased the relative heading up and affected the flow rate in the stream. Different cases were studied to improve the negative hydraulic effects resulting from the use of SB. The effect of increasing the biofilter length on the hydraulic properties of the stream was tested using different biofilter lengths (0.4, 0.8, 1.2, 1.6, 2.0 m). The results of this case showed that increasing the length increased the upstream water depth (h_1_) and relative heading up (h_1_/h_2_). In the second case, a trial was done to assess the effect of dividing the biofilter in the flow direction. Two scenarios were tested in this case: a continuous and fragmented biofilter. The results showed that the fragmented biofilter increased the upstream water depth and the relative heading up due to increasing the length in the flow direction, which matches with the first case. The effect of the biofilter height was tested in case three. Different heights (0.52, 0.26, 0.13 m) of SB were tested with a fixed length and constant flow rate. The results indicated that the relative heading up decreased when the biofilter height decreased. Thus, in order to reduce the relative heading up, the height of the biofilter should be decreased. Case four tested the effect of the biofilter with a fixed volume and constant flow rate on the relative heading up. In this case, three scenarios were tested including changing the length and height of the biofilter where the volume of the biofilter was fixed and the flow rate was constant. The results showed that for a fixed volume and constant flow rate, the relative heading up (h_1_/h_2_) decreased when the biofilter height decrease and the length increased.

Based on the results of the above cases, it can be concluded that increasing the biofilter length could enhance the removal of pollutants from streams but the hydraulic characteristics of the stream are affected as the upstream water depth and relative heading up increase. To overcome the negative impact of the SB on the hydraulic properties, the height of the biofilter should be decreased.

## 6. Conclusions

A shortage of freshwater supplies has contributed to the use of unconventional water resources in regions suffering from water scarcity. This includes the use of agricultural drain water to fill the gap between water demand and supply. Water in agricultural drains is contaminated with different sources of pollution, which require treatment before use. One of the used methods to enhance the drains’ self-purification capacity is the use of SB. The use of such filters improves the removal of pollutants but affects the hydraulic properties of the stream as it increases the upstream water level and relative heading up. The current study was carried out to assess the hydraulic effects of SB on water streams and how to improve it. From the experimental work, an empirical equation was developed to calculate the flow rate through the biofilter. The experimental results were compared with three empirical equations, but the Fadhil formula gave the closest representation to predict the flow rate through the biofilter compared with the modified Fadhil formula and Depuit formula. The results of this formula indicated that the flow rate through the biofilter depends on the upstream water depth (h_1_) more than on the relative heading up (h_1_/h_2_). Different cases were studied to test the impact of the biofilter characteristics on the hydraulic properties including the SB length, dividing SB in the flow direction (fragmented biofilter), the height of SB, and the volume. The results from case one and two indicated that increasing the biofilter length could increase the upstream water depth and the relative heading up. Increasing the biofilter length from 0.8 to 2.0 m increased the upstream water depth from 0.59 to 0.6 m and increased the relative heading up from 1.029 to 1.052. However, cases three and four revealed that decreasing the biofilter height could improve the hydraulic properties of the stream. Decreasing the biofilter height from 0.52 to 0.26 and 0.13 m decreased the upstream water depth from 0.536 to 0.625 and 0.52 m, respectively, and decreased the relative heading up from 1.03 to 1.009 and 1.0, respectively. The change of the heading up seems to be small, and this is due to the small dimensions of the canal, but these values will be larger if measured in a large-0scale canal. The use of SB could help to improve the water quality in polluted streams but lead to an increase of the relative heading up, which can be reduced by decreasing the height of the biofilter.

## Figures and Tables

**Table 1:** The operational conditions applied through the experimental study.

Runs	Length (m)	Flow Rate (L·s^−1^)	Measured Parameters	Calculated Parameters
1	0.4	4.7 to 63.3	h_1_, h_2_	Relative heading up (h_1_/h_2_)
2	0.8	2.0 to 63.7		
3	1.2	7.2 to 62.1		
4	1.6	2.8 to 67.6		
5	2	2.3 to 62.5		

**Table 2:** The measured flow rate, water depths upstream and downstream of the biofilter, the resulting relative heading up (h_1_/h_2_), and the flow rate calculated from the Dupuit formula, Fadhil equation, and modified Fadhil’s equation.

No.	L (m)	Measured Flow Rate (m^3^·s^−1^)	Upstream Water Depth (h_1_) m	Downstream Water Depth (h_2_) m	Relative Heading Up (h_1_/h_2_)	Dupuit Qcal (m^3^·s^−1^)	Fadhil Qcal (m^3^·s^−1^)	Modified Fadhil Qcal (m^3^·s^−1^)
1	0.4	0.007	0.481	0.480	1.002	0.002	0.018	0.031
2		0.016	0.509	0.505	1.008	0.008	0.023	0.034
3		0.029	0.545	0.535	1.019	0.021	0.031	0.038
4		0.035	0.559	0.548	1.020	0.024	0.035	0.039
5		0.041	0.580	0.561	1.034	0.043	0.041	0.046
6		0.045	0.591	0.570	1.037	0.048	0.045	0.047
7		0.048	0.602	0.580	1.038	0.051	0.049	0.048
8		0.052	0.610	0.585	1.043	0.059	0.052	0.051
9		0.056	0.617	0.590	1.046	0.064	0.054	0.052
10		0.060	0.627	0.598	1.048	0.070	0.058	0.054
11	0.8	0.014	0.508	0.501	1.014	0.007	0.020	0.023
12		0.020	0.526	0.516	1.019	0.010	0.023	0.024
13		0.032	0.560	0.541	1.035	0.021	0.031	0.029
14		0.038	0.578	0.552	1.047	0.029	0.035	0.033
15		0.041	0.593	0.561	1.057	0.036	0.040	0.037
16		0.046	0.606	0.569	1.065	0.043	0.044	0.041
17		0.047	0.617	0.575	1.073	0.049	0.047	0.045
18		0.052	0.628	0.583	1.077	0.053	0.051	0.047
19		0.055	0.639	0.589	1.085	0.060	0.056	0.051
20		0.062	0.657	0.600	1.095	0.070	0.063	0.057
21	1.2	0.021	0.526	0.515	1.021	0.007	0.021	0.019
22		0.027	0.543	0.527	1.030	0.011	0.025	0.021
23		0.032	0.568	0.540	1.052	0.020	0.030	0.027
24		0.040	0.588	0.551	1.067	0.028	0.035	0.032
25		0.045	0.610	0.563	1.083	0.036	0.042	0.038
26		0.047	0.620	0.570	1.088	0.039	0.045	0.040
27		0.048	0.635	0.578	1.099	0.045	0.050	0.045
28		0.054	0.650	0.585	1.111	0.053	0.055	0.052
29		0.058	0.662	0.590	1.122	0.059	0.060	0.059
30		0.065	0.680	0.600	1.133	0.067	0.068	0.066

**Table 3:** The measured flow rate and water depths upstream and downstream of the biofilter and the resulting relative heading up (h_1_/h_2_) for different SB lengths.

L (m)	Measured Flow Rate (L·s^−1^)	Upstream Water Depth (h_1_) m	Downstream Water Depth (h_2_) m	Relative Heading Up (h_1_/h_2)_
0.4	4.70	0.475	0.473	1.004
	19.20	0.519	0.513	1.012
	27.40	0.540	0.533	1.013
	36.30	0.560	0.551	1.016
	42.60	0.580	0.569	1.019
	47.70	0.590	0.578	1.021
	52.30	0.596	0.583	1.022
	57.90	0.605	0.592	1.022
	59.60	0.609	0.595	1.024
	63.30	0.6123	0.598	1.025
0.8	2	0.464	0.461	1.007
	14.10	0.506	0.501	1.010
	18.70	0.522	0.514	1.016
	23.30	0.535	0.523	1.023
	35.60	0.567	0.551	1.029
	46.20	0.590	0.573	1.030
	51.50	0.600	0.582	1.031
	53.10	0.603	0.585	1.031
	60.40	0.615	0.596	1.032
	63.70	0.620	0.601	1.032
1.2	7.20	0.483	0.479	1.008
	11.90	0.5	0.495	1.010
	18.50	0.520	0.511	1.018
	25.60	0.543	0.530	1.025
	33.60	0.561	0.543	1.033
	37.00	0.572	0.553	1.034
	41.10	0.584	0.564	1.035
	44.00	0.291	0.570	1.037
	47.30	0.598	0.576	1.038
	51.20	0.604	0.582	1.038
	53.90	0.608	0.585	1.039
	57.50	0.614	0.591	1.039
	59.60	0.618	0.594	1.040
	62.1	0.621	0.597	1.040
1.6	2.80	0.466	0.462	1.009
	6.90	0.482	0.477	1.01
	13.90	0.508	0.499	1.018
	20.30	0.531	0.517	1.027
	28.00	0.551	0.532	1.036
	34.30	0.570	0.546	1.044
	39.70	0.582	0.557	1.045
	43.30	0.592	0.565	1.048
	45.10	0.600	0.572	1.049
	48.50	0.605	0.577	1.049
	57.1	0.620	0.590	1.051
	67.60	0.634	0.603	1.051
2	2.30	0.470	0.467	1.006
	15.50	0.520	0.505	1.030
	20.60	0.536	0.515	1.041
	26.20	0.553	0.529	1.045
	33.60	0.570	0.545	1.046
	37.60	0.582	0.555	1.049
	44.00	0.591	0.563	1.050
	46.20	0.600	0.570	1.053
	48.10	0.607	0.576	1.054
	50.80	0.612	0.580	1.055
	53.50	0.618	0.585	1.056
	62.50	0.631	0.597	1.057

**Table 4:** The measured water depths upstream and downstream of the biofilter and the resulting relative heading up (h_1_/h_2_) for the continuous and fragmented biofilter.

L (m)	Flow Rates (L·s^−1^)	Case (1)			Case (2)		
		Upstream Water Depth (h_1_) m	Downstream Water Depth (h_2_) m	Relative Heading up (h_1_/h_2_)	Upstream Water Depth (h_1_) m	Downstream Water Depth (h_2_) m	Relative Heading Up (h_1_/h_2_)
0.8	47	0.59	0.573	1.029	0.595	0.577	1.031
1.2	34.3	0.561	0.543	1.033	0.565	0.545	1.036
1.6	48.47	0.605	0.577	1.048	0.608	0.579	1.050
2	46.22	0.6	0.57	1.052	0.607	0.574	1.057

**Table 5:** The measured water depths upstream and downstream of the biofilter and the resulting relative heading up (h_1_/h_2_) for different heights.

Cases	L (m)	H (m)	Upstream Water Depth (h_1_) m	Down Stream Water Depth (h_2_) m	Relative Heading Up (h_1_/h_2_)
Case (1)	0.52	1.6	0.536	0.52	1.03
Case (2)	0.26	1.6	0.525	0.52	1.009
Case (3)	0.13	1.6	0.52	0.52	1

**Table 6:** The measured water depths upstream and downstream of the biofilter and the resulting relative heading up (h_1_/h_2_) for a constant volume and variable length and height.

Cases	L (m)	H (m)	Upstream Water Depth (h_1_) m	Down Stream Water Depth (h_2_) m	Relative Heading Up (h_1_/h_2_)
Case (1)	0.4	0.52	0.525	0.52	1.0096
Case (2)	0.8	0.26	0.521	0.52	1.0019
Case (3)	1.6	0.13	0.52	0.52	1

## Data Availability

Not applicable.

## References

[1] El-Nashar W.Y., Elyamany A.H. (2018). Managing risks of the Grand Ethiopian renaissance dam on Egypt. Ain Shams Eng. J..

[2] Abd El Moniem A.A. (2009). Overview of water resources and requirements in Egypt; the factors controlling its management and development. J. Environ. Stud..

[3] Abd-Elhamid H.F., Abd-Elmoneem S.M., Abdelaal G.M., Zeleňáková M., Vranayova Z., Abd-Elaty I. (2021). Investigating and Managing the Impact of Using Untreated Wastewater for Irrigation on the Groundwater Quality in Arid and Semi-Arid Regions. Int. J. Environ. Res. Public Health.

[4] AbdEllah R.G. (2020). Water resources in Egypt and their challenges, Lake Nasser case study. Egypt. J. Aquat. Res..

[5] Ibrahim A.I. (2017). Impact of Ethiopian Renaissance Dam and population on future Egypt water needs. Am. J. Eng. Res. (AJER).

[6] Abdelhafez A.A., Metwalley S.M., Abbas H.H., Omran E.S., Negm A. (2020). Irrigation: Water Resources, Types and Common Problems in Egypt. Technological and Modern Irrigation Environment in Egypt.

[7] Dakkak A. (2014). Egypt’s Water Crisis–Recipe for Disaster. Retrieved Febr..

[8] Lamei A., Van der Zaag P., Von Muench E. (2008). Impact of solar energy cost on water production cost of seawater desalination plants in Egypt. Energy Policy.

[9] Elbana T.A., Bakr N., Elbana M. (2017). Reuse of treated wastewater in Egypt: Challenges and opportunities. Unconventional Water Resources and Agriculture in Egypt.

[10] El Gamal F., Mostafa H., Shalby A., Hamdy A., El Gamal F., Lamaddalena N., Bogliotti C., Guelloubi R. (2005). Reuse of low quality water in Egypt. Non-Conventional Water Use: WASAMED Project.

[11] El-Agha D.E., Molden D.J., Ghanem A.M. (2011). Performance assessment of irrigation water management in old lands of the Nile delta of Egypt. Irrig. Drain. Syst..

[12] Abdel-Rahman W.H. (2002). Discharge of Domestic Waste Water into Rivers. Ph.D. Thesis.

[13] Ramírez-Baca N., Saucedo-Terán R., Manzanares-Papayanopoulos L.I., Carrazco-Palafox J., Nevárez-Moorillón G.V. (2005). Treatment for small polluted rivers: Design and performance of an experimental structure. Water S. Afr..

[14] Moussa M.S., Salem H., El Gammal M.A., Atta M.A., Abdel Khalek M.A. Enhancement of the self-purification in the West Bank Drains. Proceedings of the 2nd International Conference Environmental Sciences and Technology.

[15] El Monayeri D.S., Atta N.N., El Mokadem S., Abou El-fotoh A.M. Biological Treatment of Drain’s Water Using Submerged Bioreactors. Proceedings of the Second Ain Shams University International Conference on Environmental Engineering.

[16] El-Monayeri D.S., El-Karamany H., El-Gohary E.H. (2003). Case Study for Bilbeas and El-Qalyoubia Drains.

[17] El Monayeri D.S., Atta N.N., El Mokadem S., EL-Gohary E.H. Enhancement of Bilbeas Drain Water Quailty Using Submerged Biofilters (SBs). Proceedings of the Eleventh International Water Technology Conference, IWTC11 2007.

[18] He S., Li J., Peng Z., Zhang Q., Xu Y., Yang H. (2011). Nitrobacteria community structure during the startup in a submerged biofilters for in-situ remediation of contaminated stream. Fresenius Environ. Bull..

[19] Abdel Daiem M.M., Hatata A., El-Gohary E.H., Abd-Elhamid H.F., Said N. (2021). Application of an artificial neural network for the improvement of agricultural drainage water quality using a submerged biofilter. Environ. Sci. Pollut. Res. Int..

[20] Salem R. (2016). Monitoring of Pollutants in Waste Water in Some Egypt Drains. J. Plant Prot. Path. Mansoura Univ..

[21] Fadhil M., Al-Mohammed M., Mohammed S.H. (2015). Flow through and over gravel gabion weirs. J. Kerbala Univ..

[22] Mulqueen J. (2005). The flow of water through gravels. Ir. J. Agric. Food Res..

[23] Khalifa A., Abdelmonaem Y.K., El-azezy E. (2003). The Hydraulic Proprieties of Flow through the Horizontal Bioreactors.

[24] Bowles J.E. (1979). Physical and Geotechnical Prosperities of Soils.

[25] Mohamed H.I. (2010). Flow over Gabion Weirs. J. Irrig. Drain. Eng. ASCE.

[26] Muslu Y. (1986). Distribution of Residence Times in Biological Filters. II: Experimental Confirmation. J. Environ. Eng..

[27] Parker D.S. Research Needs for Trickling Filter Design: A Consultant’s Perspective. Proceedings of the International Conference on Fixed-Film Biological Processes, 2nd.

[28] Poon C.P., Scholze R.J., Bandy J.T., Smith E.D. (1984). Upgrading Army Sewage Treatment Plant Trickling Filters with Synthetic Media.

[29] Logan B.E., Hermanowicz S.W., Parker D.S. (1987). A fundamental model for trickling filter process design. J. Water Pollut. Control Fed..

